# Caseload, clinical spectrum and economic burden of infectious diseases in patients discharged from hospitals in Germany

**DOI:** 10.1007/s15010-025-02507-x

**Published:** 2025-04-09

**Authors:** H. Stocker, F. Kron, P. Hartmann, K. de With, M. Addo, M. Vehreschild, G. Fätkenheuer, B. Salzberger, L. E. Sander, Jan Rupp

**Affiliations:** 1 Department of Infectious Diseases, St. Joseph Hospital, Berlin-Tempelhof, Berlin, Germany; 2 Department of Infectious Diseases, Respiratory and Critical Care Medicine, Charité - Universitätsmedizin Berlin, Berlin, Germany; 3 Department I of Internal Medicine, University Hospital of Cologne, University of Cologne, Cologne, Germany; 4 VITIS Healthcare Group, Cologne, Germany; 5 FOM University of Applied Sciences, Essen, Germany; 6 Department of Infectious Diseases, Hospital of the Cellites - St. Vinzenz, Cologne, Germany; 7 Institute of Infectious Diseases, University Hospital Carl Gustav Carus at the Technical University, Dresden, Germany; 8 German Center for Infection Research, University Medical Center Hamburg-Eppendorf, Hamburg, Germany; 9 Department 2 of Internal Medicine, Infectious Diseases, Goethe University Frankfurt, University Hospital, Frankfurt am Main, Germany; 10 Department I of Internal Medicine, Division of Infectious Diseases, Medical Faculty, University Hospital of Cologne, University of Cologne, Cologne, Germany; 11 Department of Infection Prevention and Infectious Diseases, University Hospital Regensburg, Regensburg, Germany; 12 Institute of Medical Microbiology, University Hospital Schleswig-Holstein/ Campus Lübeck and Kiel, Kiel, Germany; 13 Infectious Disease Clinic, University of Lübeck and University Hospital Schleswig-Holstein/ Campus Lübeck, Lübeck, Germany

**Keywords:** Infectious diseases, Health economic analysis, Antimicrobial resistance, Hospitalization and caseload, Healthcare system and specialization, Cost and reimbursement, Healthcare system and specialization

## Abstract

**Background:**

Over the last century infectious diseases have been kept under control in industrialized countries thanks to advances in hygiene, prevention and antimicrobial treatments. However, the emergence of HIV, the COVID-19 pandemic, and the rise of resistant bacteria exemplify that infectious diseases continue to pose a global threat. A comprehensive understanding of the caseload, spectrum of infectious diseases and the economic impact they pose is required to develop strategies for managing infectious diseases in a resilient healthcare system.

**Objectives:**

(i) to determine the proportion of adult patients discharged from German hospitals with primary diagnoses classified as an infectious disease, (ii) to describe the clinical spectrum of these diagnoses, case characteristics, and hospital settings, and (iii) to estimate the total economic burden that these cases contribute to the in-patient sector of the healthcare system.

**Methods:**

A retrospective case-control study was performed using publicly available data on ICD10 codes assigned as primary diagnoses, case characteristics, treatment settings, and cost weights from all patients discharged from German hospitals in 2022.

**Results:**

1,728,824 adult patients (12% of all adult patients) were discharged with a primary diagnosis classified as an infectious disease. They were assigned 912 individual ICD10 codes. The 15 and 79 most frequently used codes comprised 40% (top 40% ID population) and 80% (top 80% ID population) of all infectious disease cases, respectively. In the top 80% ID population, patients were older, were more likely to be male, and had higher complexity and comorbidity levels than the reference population, which consisted of all adult patients minus the patients in the top 80% ID population. The mean length of stay of patients forming the top 80% ID population was 8.0 days vs. 6.1 days in the reference population. The median (IQR) cost weight was 0.663 (0.544–1.030) translating into €2,541 per case.

**Conclusions:**

In Germany, patients with infectious diseases constitute a significant proportion of all inpatients, with a broad spectrum of conditions. These patients are generally older, more severely ill, and require longer hospital stays than those without a primary infectious disease diagnosis, contributing substantially to the overall economic burden on the healthcare system.

**Supplementary Information:**

The online version contains supplementary material available at 10.1007/s15010-025-02507-x.

## Background

The control of infectious diseases has been a cornerstone of modern medicine’s success [1]. With the introduction of basic hygiene concepts and the discovery of antimicrobials, it initially seemed that infectious diseases had been largely defeated. However, this optimism proved to be short-lived. The assumption that infectious diseases could be held in check through the widespread use of antimicrobials had dire consequences: The excessive and uncontrolled use of these agents facilitated the emergence of resistant microorganisms, severely limiting today’s treatment options for both common and rare infections. Moreover, outbreaks and epidemics caused by drug-resistant and emerging pathogens impose a considerable economic burden on healthcare systems.

While clinical conditions such as pneumonia and urinary tract infections remain the same, the pathogens responsible for these infections are constantly evolving. This necessitates the timely and ongoing development of diagnostic and therapeutic strategies, accompanied by continuous advancements in the expertise of infectious disease (ID) specialists. In response to this challenge, most countries have established “Infectious Diseases” as an independent clinical specialty within their healthcare systems. This has shown to be beneficial both for the quality of care and the resilience of their healthcare systems. In contrast, the clinical specialty of Infectious Diseases had been only minimally represented within the mosaic of Germany’s healthcare system. However, in 2021, the country introduced a formal educational program for physicians aiming for board certification in Infectious Diseases, marking the beginning of a concerted effort to expand this expertise across hospitals in the years to come.

Hospital resources for inpatient treatment should ideally be allocated based on the spectrum and severity of diseases in different specialties. The current DRG system in Germany has been developed before specialty services in infectious diseases were established in 2021, thus neither ID expertise nor the economic impact of specialized medical care for infectious diseases are represented in the current reimbursement system. Successfully implementing infectious disease units within the existing healthcare framework, therefore, requires a thorough understanding of the current state of the system.

To this end, we conducted an analysis based on data from Germany, representing a developed healthcare system in a moderate climate zone. We specifically analyzed: (i) the caseload of inpatients with first-listed infectious disease diagnoses compared to all hospitalized patients, (ii) the demographics of these patients, (iii) the clinical spectrum of infectious disease cases, and (iv) the economic impact of these patients, including factors such as patient complexity, hospital distribution, and trusteeship, and cost-weight.

## Materials and methods

This is a retrospective case control study conducted on publicly available data sources that document all DRG settled discharge cases (i.e. all cases excluding patients with psychiatric first listed diagnoses) from all German hospitals in 2022. The terms “cases” and “patients” are used interchangeably throughout this article.

### Definition of terms

#### Cost weight

The cost weight is an indirect measure of care expenditure for cases clustered within an individual DRG. Using the Disease Related Group (DRG) algorithm devised by the federal Institute for the Hospital Remuneration System (InEK), cases are classified into a group (i.e. a DRG) based on their primary and secondary diagnoses, medical procedures, and patient-related characteristics. A single DRG may house cases with several different primary diagnoses but similar expenditures. This system ensures that cost weight relations between DRGs are fair. In 2022, a cost weight of 1.000 corresponded to 3,833.00 €. Additional information on cost weights can be found in the supplementary appendix.

#### Virtual cost weight

Because individual ICD10 codes cannot be directly assigned a cost weight we backcalculated a virtual cost weight for each ICD10 code comprised in the top 80% ID population (see below).

Each ICD10 code that was included in the top 80% ID population was individually submitted to the aG-DRG-Report-Browser 2022 (data source b) to identify all the DRGs that were triggered by each code. The number of cases of the respective code within each DRG were then multiplied by the case weight of the respective DRG yielding a case mix (see below) for the ICD10 code per DRG. This operation was performed for each of the DRGs that were triggered by the respective code and the resulting case mixes were summed up. This sum was then divided by the total number of cases of the respective ICD10 code. By this algorithm we could determine an average case weight (or a price tag) for each ICD10 code termed here as the virtual case weight.

Additional information on virtual cost weights can be found in the supplementary appendix.

#### Case-mix (CM)

The case-mix is the sum of the cost weights of all DRGs provided within a period.

#### Patient clinical complexity level (PCCL)

The PCCL is calculated with an algorithm that is fed by patient characteristics and secondary diagnoses. It indicates the severity of the complication or comorbidity (CC) yielding results ranging from 0 (no clinical complexity) to 6 (most severe clinical complexity).

#### Inliers and outliers

In the DRG framework, the term “inliers” characterizes those cases whose length of stay (LOS) is within the expected LOS range, that was derived from the majority of cases within a specific DRG. For inliers, hospital expenses and reimbursements are largely equivalent. In contrast, outliers represent patient cases that deviate from the expected LOS within a DRG. Outliers are characterized in low and high outliers whether they fall below the lower or exceed the higher DRG boundary, respectively. High outliers therefore strain the hospitals’ budgets, while low outliers may increase the hospitals’ profits.

### Data sources

Data were collected from two publicly available sources:

- *InEK-Datenbrowser (data source a)*: It provided aggregated data from all registered hospitals in Germany [2].
- *aG-DRG-Report-Browser (data source b)*: It provided data from selected hospitals, termed “InEK-Kalkulationskrankenhäuser”, that allow a case specific cost calculation for each DRG across hospitals [3].

### Selection of cases and references (Fig. 1)

#### Step 1: selection of the baseline population

Inclusion criteria (all of the following):

1. Any patient discharged from any German hospital in 2022.
2. Age ≥ 18 years.

#### Step 2: selection of the population with a primary diagnosis classified as infectious disease (total ID population)

Inclusion criteria (all of the following):

1. Any patient discharged from any German hospital in 2022.
2. Age ≥ 18 years.
3. Patients discharged with an ICD10 code classified as an infectious disease.

For the latter criterion, five ID physicians independently assessed all ICD10 codes (*n* = 8,449) assigned as primary diagnoses to cases in the baseline population and classified them according to the presence or absence of an infectious disease relevant to in-patient care. The results of the individual assessments were combined. The board then reevaluated the divergent results and reached a consensus.

The total ID population was used (i) to determine the absolute number and proportion of cases with primary diagnoses classified as IDs, and (ii) to subclassify the ICD10 codes (Step 3).

#### Step 3: subclassification of infectious diseases

ICD10 codes of primary diagnoses classified as IDs were subclassified into the following mutually exclusive groups:

1. Infections of the CNS: (meningitis, encephalitis, myelitis, brain abscess).
2. Infections of the lower respiratory tract: (tracheitis, bronchitis, bronchiolitis, exacerbation of COPD triggered by infection, pneumonia, infections caused by SARS-CoV-2, influenza / parainfluenza viruses, RSV, metapneumovirus, *B. pertussis*, lung abscess, infections of the mediastinum and the pleural space).
3. Infections of the gastrointestinal tract and the peritoneum: (esophagitis, gastroenteritis, enteritis, colitis, enterocolitis, food poisoning, diverticulitis, appendicitis, anal / rectal abscess, cholecystitis, cholangitis, liver abscess, hepatitis (acute and chronic), peritonitis, intraabdominal abscess).
4. Infections of soft tissues (infections of the skin, subcutaneous tissues, muscles, tendons, lymphatics, wounds).
5. Infections of the urinary tract: (cystitis, pyelonephritis, interstitial nephritis, renal abscess, perinephric abscess, infections of the urethra, infections of the prostate gland).
6. Infections of bones and joints (arthritis, osteomyelitis, osteitis, spondylarthritis, spondylodiscitis, discitis, prosthetic joint infections and infections related to implants for osteosynthesis, intraspinal, extradural and subdural abscesses).
7. Infections of the heart, mediastinum and pleural space (endocarditis, myocarditis, pericarditis, mediastinitis, pleural empyema, pyothorax).
8. Sepsis, toxic shock syndrome.
9. Tuberculosis and other mycobacterioses.
10. HIV infection and Immune Reconstitution Inflammatory Syndrome (IRIS): (excluding pneumocystosis, toxoplasma encephalitis, cryptococcosis, and other opportunistic diseases coded elsewhere)
11. Tropical diseases: (malaria, dengue, hemorrhagic fever, typhoid fever, rickettsioses, leptospirosis, schistosomiasis, leishmaniasis, mycetoma)
12. Zoonotic diseases: (brucellosis, Q-fever, relapsing fever, tularemia, tick borne encephalitis, erysipeloid, listeriosis, salmonellosis, infections by *Pasteurella* spp. and *B. henselae*)

#### Step 4: identification of the most frequently used ICD10 codes as primary diagnoses for cases with an infectious disease

Cases with ICD10 codes classified as IDs were sorted in descending order based on the number of cases per ICD10 code. The top 40% and 80% ID populations identify the ICD10 codes that comprised 40% and 80% of the most frequent cases per ICD10 codes, respectively. Truncations of the dataset (see supplementary appendix) forced us to limit this part of our analysis to the codes that contained in sum 80% of the most frequent cases with a primary ID diagnosis. The rationale for subdividing this population into the top 40% and the top 41–80% ID populations was the simple fact that these two populations represent the lower and the upper half of the cases that were accessible to analysis. The top 80% ID population was further analyzed for demographics, length of stay (LOS), Patient Complexity and Comorbidity Level (PCCL), size and trusteeship of the attending hospital as well as reimbursements.

The top 40% ID population was subclassified according to diagnoses that could be attributed solely to cases that can be managed in internal medicine departments - in contrast to cases that require surgical interventions. The rationale for choosing this population was the fact that the ICD10 codes that formed this population lacked ambiguity in terms of the necessity of surgical interventions: Unlike many of the ICD10 codes used in the top 80% ID population the ICD10 codes comprising the top 40% ID population could be confidently classified according to whether surgery was likely to be part of the case management or not.

The top 41–80% ID population identifies all cases of the top 80% ID population minus the top 40% ID population. This population was used to examine the differences in cost weights between the lower and the upper half of ICD10 codes classified as IDs. The rationale for choosing this subdivision of the top 80% ID population was to assess imbalances of cost weights among ICD10 codes with high numbers of cases and ICD10 codes with lesser numbers of cases.

#### Step 5: definition of the reference population

Due to truncations of the source data, we were unable to retrieve a dataset that contains information on all cases without a primary ID diagnosis. Therefore, the top 80% ID population (*n* = 1,377,985) was defined as the major analysis population. While the reference population was defined as the remaining cases (*n* = 13,457,298). The reference population, therefore, contains all cases without primary ID diagnoses plus 350,839 cases of the bottom 20% ID population forming 2.4% of the total study population. These 350,839 cases were coded in 833 different ICD10 codes with case numbers per code ranging from 5 to 4,403.

#### Step 6: analysis of the virtual cost weight of the top 80% ID population the top 40% ID population and the top 41-80% ID population

### Statistics

Data were analyzed using MS Excel (Microsoft Corporation, Redmond, Washington, USA). Descriptive analyses were performed comparing the top 80% ID population and the reference population based on data from data source (a) Both populations were compared with respect to the following items: age, sex, length of stay in hospital, size and trusteeship of hospitals, and patient clinical complexity level (PCCL)- structure. For additional DRG-related analyses of case-mix (CM) and case-mix index (CMI) data were retrieved from data source (b) Multiplication of the base case value with the determined CMI was performed to estimate the overall budget volume of ID cases.

## Results

### Caseload

In total, 14,835,283 inpatients, aged ≥ 18 years, were discharged from German hospitals in 2022 (baseline population). 1,728,824 patients with primary diagnoses classified as an ID (total ID population) were discharged, corresponding to 12% of all patients. Approximately 40.7 million (28%) of all secondary diagnoses classified as an ID (data not shown). Patients with primary diagnoses classified as an ID were assigned 912 individual ICD10 codes. The number of cases per code ranged from 147,137 (N39.0 Urinary tract infection, site not specified) to 5 (M86.49 Chronic osteomyelitis with draining sinus, site not specified).

The subclassification of ICD10 codes assigned to cases forming the total ID population yielded the following results (Fig. 2): 468,524 (27.1%) patients with infections of the lower respiratory tract, 335,362 (19.4%) with infections of the gastrointestinal tract and peritoneum, 225,011 (13.0%) with infections of the urinary tract, 207,604 (12.0%) with infections of soft tissues, 62,783 (3.6%) with infections of bones and joints, 33,401 (1.9%) with infections of the heart, mediastinum and pleural space and 25,996 (1.5%) with CNS infections. Independently of the former patients, 64,027 (3.7%) patients with an ICD10 code including the term “sepsis” or “toxic shock” were recorded. Less than 1% (6,684) of patients were assigned a code that included tuberculosis and other mycobacterial infections, 1,267 (0.1%) with codes that comprised tropical diseases, 992 (0.1%) with codes that contain the terms “HIV” or “IRIS”, and 809 (0.1%) patients with codes that comprised zoonotic diseases. The remaining 17.1% of patients could not be subclassified into any of the above-mentioned categories.

A total of 700,035 (40%) patients were assigned one of the 15 most frequently used ICD10 codes (top 40% ID population). 1,377,985 (80%) patients were assigned one of the 79 most frequently used ICD10 codes (top 80% ID population). The bottom 20% of ID cases were scattered over 833 different ICD10 codes with case numbers per code ranging from 5 to 4,403. Codes related to HIV / IRIS, tuberculosis / mycobacterial infections, zoonotic diseases and tropical infections were not amongst the top 80% ID population.

### Case characteristics

Case characteristics, which could only be derived for the top 80% ID population are shown in Table 1; Fig. 3. Patients forming this population were slightly more frequently male, older, more complex, and had more comorbidities than the patients forming the reference population. Along the same line, the mean length of stay was longer than that in the reference population (8.0d vs. 6.1d), and the inliers and high outliers exceeded the respective numbers in the reference population by 7.5% and 3.0%.

**Table 1:** Case characteristics

	Top 80% ID population	Reference population
Cases [n]	1,377,985	13,457,298
Cases [%]	9	91
Cases per gender		
female [%]	47	53
Male [%]	53	47
Diverse [%]	0	0
Cases per age group		
18-49 [%]	21	24
50-79 [%]	47	53
≥80 [%]	32	23
Cases per length of stay		
Length of stay (mean) [d]	8.0	6.1
Length of stay [SD]	8.8	na
Low outliers [%]	9.5	20.0
inliers [%]	81.5	74.0
High outliers [%]	9.0	6.0
Cases per PCCL		
0 [%]	45	65
1 [%]	15	13
2 [%]	15	9
3 [%]	17	8
4 [%]	7	3
5 [%]	1	1
6 [%]	0	0
Cases per hospital size [number of beds]		
≥1.000 [%]	12	16
800-999 [%]	5	6
600-799 [%]	12	12
400-599 [%]	22	21
200-399 [%]	32	28
<200 [%]	16	16
Cases per hospital size relative to total cases per hospital size [number of beds]		
≥1.000 [%]	7	93
800-999 [%]	8	92
600-799 [%]	9	91
400-599 [%]	10	90
200-399 [%]	10	90
<200 [%]	10	90
Cases per hospital trusteeship relative to total cases per hospital trusteeship		
Public / non-profit [%]	9	91
Private [%]	9	91
Virtual cost weight per ICD10 code		
Minimum	0.332	
Q1	0.544	
Median	0.663	
Q3	1.030	
Maximum	4.967	

### Hospital size and trusteeship

Overall, most patients in the top 80% ID population were discharged from smaller hospitals (< 400 beds), with almost 50% of all patients in this population being discharged from respective hospitals as opposed to 44% of the reference population. Related to the totally available beds in the respective hospital size categories, approximately 4.4 ID patients per bed had been discharged from hospitals with 150–199 beds in contrast to 3.1 ID patients from hospitals with > 800 beds (Fig. 4). With respect to hospital trusteeship, public/nonprofit and private hospitals did not differ in terms of the proportion of patients with a primary ID diagnosis in their total caseload.

### Internal medicine versus surgery

Within the top 40% ID population 625,053 (89%) patients were assigned a diagnosis that was classified as related to internal medicine: 258,268 (37%) respiratory tract infections, 147,137 (21%) urinary tract infections, 126,857 (18%) gastrointestinal infections (non-surgical), 61,273 (9%) soft tissue infections, and 31,518 (5%) other infections (internal medicine). 74,982 (11%) cases were assigned a diagnosis related to specialties other than internal medicine (Table 2).

**Table 2:** Spectrum of cases within the top 40% ID population

ICD10 code	Full description	Cases / ICD10 [*n*]	Cumulative cases [*n*]	Cumulative cases [%]
N39.0	Urinary tract infection, site not specified*	147,137	147,137	9
J12.8	Other viral pneumonia*	93,561	240,698	14
A46	Erysipelas*	61,273	301,971	17
K57.32	Diverticulitis of large intestine without perforation or abscess without bleeding*	51,871	353,842	20
A09.9	Other and unspecified gastroenteritis and colitis of unspecified origin*	45,913	399,755	23
J18.1	Lobar pneumonia, unspecified organism*	41,805	441,560	26
J18.9	Pneumonia, unspecified organism*	41,372	482,932	28
B99	Other and unspecified infectious diseases*	31,518	514,450	30
J22	Unspecified acute lower respiratory infection*	30,916	545,366	32
A09.0	Other and unspecified gastroenteritis and colitis of infectious origin*	29,073	574,439	33
J44.09	Chronic obstructive pulmonary disease with acute lower respiratory infection FEV unspecified*	26,962	601,401	35
K57.22	Diverticulitis of large intestine with perforation and abscess, without bleeding#	25,450	626,851	36
K35.8	Other and unspecified acute appendicitis#	25,285	652,136	38
K35.30	Acute appendicitis with localized peritonitis, without perforation or gangrene#	24,247	676,383	39
J15.9	Unspecified bacterial pneumonia*	23,652	700,035	40
Cases allocatable to internal medicine*			625,053	89
Cases allocatable to surgery#			74,982	11

### Distribution of virtual cost weights

The median (IQR) cost weights in the top 80% ID population, the top 40% ID population and the top 41 to 80% ID population were 0.663 (0.544–1.030), 0.759 (0.576–0.813) and 0.663 (0.533–1.102), respectively (Fig. 5). It illustrates that virtual cost weights were similar throughout the spectrum of the 79 most used ICD10 codes of the top 80% ID population. In total the 1,377,985 cases of this population were reimbursed with 4,358,770,615 €.

## Discussion

In 2022, more than one out of ten adult inpatients discharged from German hospitals had a primary ID diagnosis. Furthermore, in 28% of all cases one or more IDs were listed in the secondary diagnoses. These numbers illustrate the relevance infectious diseases continue to have for health care systems of developed countries in temperate zones. Our data show a higher burden of infectious diseases than previous work conducted on all patients discharged from nine municipal hospitals in Berlin, Germany between 2009 and 2014, which found that 8.9% of patients were discharged with primary ID diagnoses [4]. This discrepancy may be explained by the lower number of ICD10 codes considered for inclusion in the former study and by the SARS-CoV-2 pandemic still ravaging during the period assessed in this study. It might also reflect the ongoing trend of moving medical treatments and care from hospital settings to outpatient care, a trend which might have had less impact on the numbers of inpatient treatment episodes of patients with infectious diseases. Patients with primary ID diagnoses were slightly older and more severely ill than cases in the reference population, as shown by the higher “Patient Level of Complexity and Comorbidity Level” (PCCL) in the ID population. Looking at the PCCL, approx. 25% of cases with primary ID diagnoses were assigned PCCL stages 3–6 in contrast to 12% of cases in the reference population. Accordingly, the mean length of stay in the ID population was considerably longer than in the reference population (8,0 vs. 6,1 days). Taken together these findings suggest, that care for patients with infectious diseases will unlikely be shifted from the inpatient to the outpatient sector and that the number of hospital beds required for such patients will need to be kept stable if not increased.

In line with the findings from the Berlin study, this study consistently shows that infections of the respiratory tract are the most common reasons for hospitalizations, accounting for 20% to approximately 30% of all ID cases. Infections of the gastrointestinal and urinary tract and soft tissues that ranked second to fourth, taken together made up 35–44% of all cases with primary ID diagnoses, confirming the results of an analysis on the epidemiology of ID hospitalizations in the United States in the Nationwide Inpatient Sample for 1998–2006 [5]. Infections of the CNS, bones and joints accounted for approximately 5–7% of cases. Only 4% of patients were discharged with ICD10 codes related to sepsis. The codes for sepsis, however, must not be used as first-listed diagnosis according to the German coding guidelines, therefore our results underestimate the burden of sepsis. A similar bias of our data applies to HIV related opportunistic diseases, in which cases the code for HIV must be listed in the secondary diagnoses.

In considering the healthcare structure in Germany, it is noteworthy that a slightly higher proportion of cases with primary infectious disease diagnoses was treated in hospitals with 400 beds or fewer, compared to those with more than 400 beds. This suggests that expertise in infectious diseases should be accessible not only in big referral hospitals but also in smaller hospitals, typically located in rural areas. This holds true for all types of hospitals across all trusteeships.

Regarding revenues and relative length of stay in hospital, the median (IQR) cost weight / case in the top 80% ID population was 0.663 (0.544–1.030), and patients in this population spent 1.9 days longer in hospital than patients in the reference population.

Based on the federal base cost value for 2022 of 3,833.07€ per cost weight point, this translates into a median (IQR) reimbursement of 2,541€ (2,085€– 3,948€) / case. In other terms, assuming a median length of stay of 8.0d the typical case with a primary ID diagnosis yielded a reimbursement of 318€ / day spent in hospital. The total case mix for this population amounts to 1,137,149 points, corresponding to a reimbursement volume of €4,358,770,615. To answer the question whether this funding is appropriate or not, we need to look at how cost weights and the standardized length of stay are currently being determined. The system to achieve this is a data driven system, which relies on current practice, rather than on standard of care. As described in the materials and methods section and the supplementary appendix, the cost weight (i.e. the price tag) of each individual DRG and the standardized length of stay are calculated each year based on information collected from representative hospitals. This procedure, however, is currently biased by the fact, that before 2022 very few hospitals with departments specialized in infectious diseases were part of this representative hospital group. Therefore, the cost weights and standardized lengths of stay of cases with primary diagnoses classified as IDs found in this study reflect a hospital landscape in which patients with infectious diseases were being treated with little input of ID specialists. Therefore, both the cost weights and the standardized lengths of stay are likely to change in the following years, if more and more hospitals with proper ID services are included in the representative hospital group.

How should inpatient care for patients with infectious diseases best be organized in the future? An answer to this question can at least in part be derived from our data: The distribution of ID categories within the top 40% ID population is largely consistent with the distribution of disease categories in the complete ID population. Therefore, the top 40% ID population can serve as a surrogate for identifying the departments that most frequently deal with ID patients. Notably, 89% of cases within the top 40% ID population were judged to be candidates for management in internal medicine departments without any need for either surgical intervention or intensive care. Notably, however, these internal medicine cases cannot be assigned to a single subspecialty of internal medicine. They rather were spread over a broad spectrum of subspecialties, which argues for centralizing care of ID patients in ID departments that closely collaborate with the other disciplines of internal medicine. The second pillar of integrated care structures for patients with infections are consultancy services. An argument for the roll-out of such services can also be found in our data: While only 79 ICD10 codes comprise 80% of the ID cases, forming the core of patients with infectious diseases, the vast majority of ICD10 codes (*n* = 833) were assigned to the remaining 20% of cases, let alone the huge number of secondary ID diagnoses. Currently, ID consultants assess only 3% of patients in German university hospitals [6]. These cases tend to have higher PCCL values, case weights, and longer-than-expected lengths of stay, suggesting a need for broader ID consultation services. Previous studies indicate that patients with higher case weights often benefit from interdisciplinary care. Specifically, ID consultation has been shown to reduce in-hospital mortality and shorten discharge times for patients with bloodstream infections, particularly those caused by *Staphylococcus aureus* [7], and improve outcomes for *Pseudomonas aeruginosa* infections through effective source control and appropriate antibiotic therapy [8].

Our study has limitations in terms of constraints in data availability and the potential impact on the accuracy of our findings: We were unable to extract detailed information regarding secondary diagnoses categorised as infectious diseases. The dataset was truncated, limiting our analysis to a population that represents only 80% of the total infectious disease population. Consequently, the reference population includes 20% of the total ID population, which may introduce a slight bias when comparing cases with first-listed diagnoses to those without.

As a result, our data likely underestimate the true caseload and economic burden of infectious diseases within German hospitals, as secondary diagnoses could not be accurately assessed. Due to coding guidelines, certain conditions, such as HIV-related infections and infections leading to sepsis, may not be fully represented within the population characterised by first-listed ICD-10 codes for infectious diseases. This issue also extends to pathogen-specific infections, including those caused by *S. aureus* and *P. aeruginosa*.

Despite these limitations, this remains the only and most comprehensive analysis of the landscape of infectious diseases in German hospitals to date, encompassing aspects of prevalence, spectrum, and economic burden.

We conclude that patients with infectious diseases account for a substantial proportion of the overall hospital caseload. The range of diseases is wide, yet most diagnoses fall under the domain of internal medicine, with no specific organ-based subspecialty emerging as the primary provider of care for these patients. The caseload is broadly distributed across hospitals of varying trusteeship, with a slight tendency towards smaller institutions.

As an increasing number of hospitals offer specialized infectious disease services and contribute data to the reimbursement system, this shift is expected to influence future remuneration policies. It may prompt revisions to the compensation framework, ensuring it more accurately reflects the complexity and resource-intensive nature of managing these patients.

**Fig. 1:**
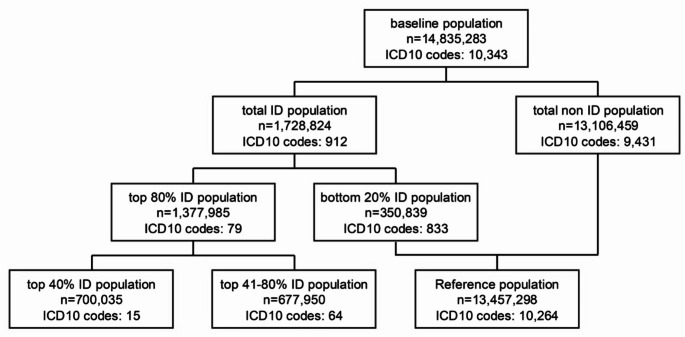
Study population. The baseline population consists of all patients discharged from German hospitals. The total ID population consists of all cases with first-listed diagnoses classified as infectious diseases. The top 80% ID population is derived by sorting the number of cases per ICD10 code in a descending order. Those cases with the highest number of cases per ICD10 code that sum up to 80% of cases of the total ID population form the top 80% ID population (i.e. 80% of patients were discharged with the most frequently used 79 first-listed ICD10 codes) The top 40% and the top 41–80% population are derived accordingly. The bottom 20% ID population contains those cases with the lowest numbers of cases per ICD10 code that sum up to 20% of cases of the total ID population. The reference population is a sum of the total non ID population and the bottom 20% ID population

**Fig. 2:**
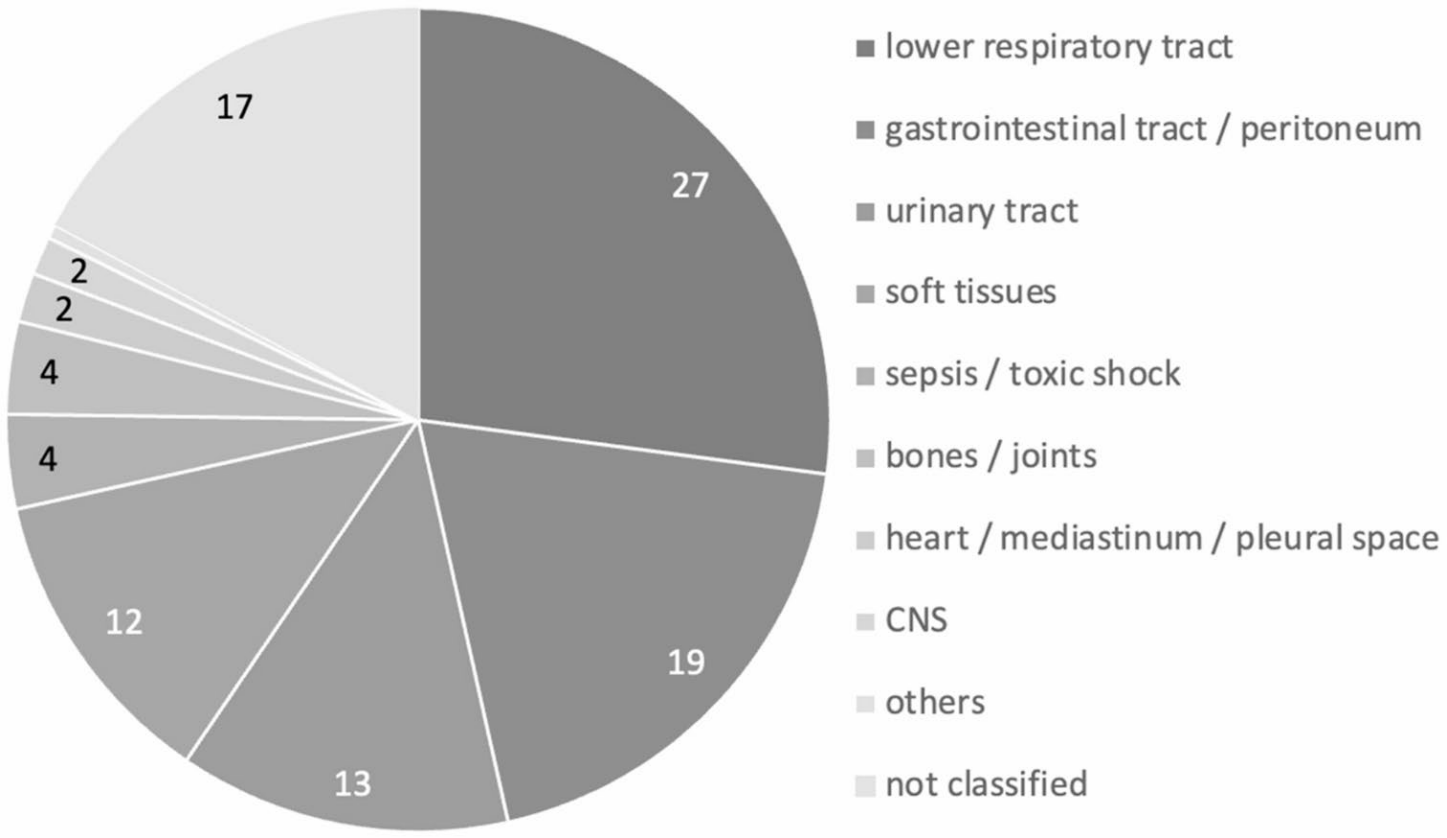
Proportion of infectious disease subcategories in the total ID population [%]. Infections of the lower respiratory tract, the gastrointestinal tract and the peritoneum, the urinary tract, soft tissues as well as sepsis and toxic shock accounted for approximately 75% of all ID cases. The subcategory “others” accounted for 0.56% of all cases with first-listed diagnoses classified as IDs. This subcategory contains cases with tuberculosis / mycobacterioses (0.39%), tropical diseases (0.07%), HIV / IRIS (0.06%), and zoonoses (0.05%)

**Fig. 3:**
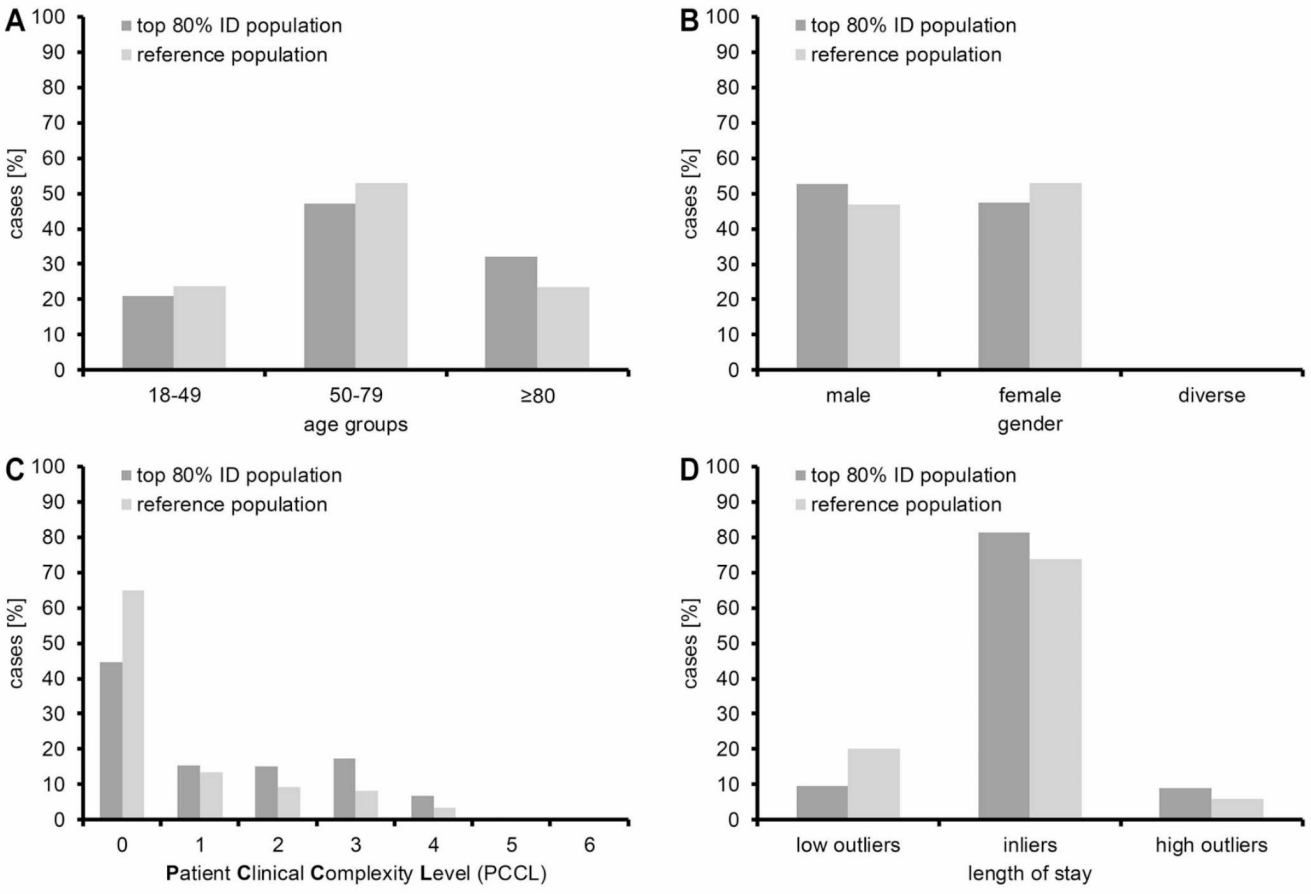
Case characteristics of the top 80% ID population compared to the reference population. Comparison of age groups (panel **A**), gender (panel **B**), PCCL (panel **C**) and length of stay (LOS) (panel **D**) of the top 80% ID population (dark grey) compared to the reference population (light grey)

**Fig. 4:**
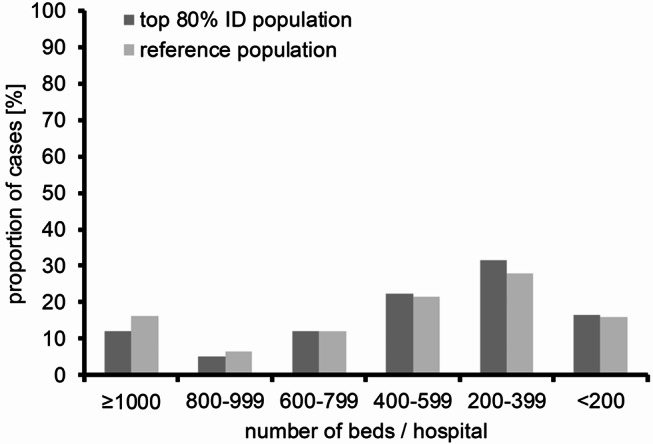
Distribution of cases across hospitals of different sizes of the top 80% ID population (dark grey) compared to the reference population (light grey)

**Fig. 5:**
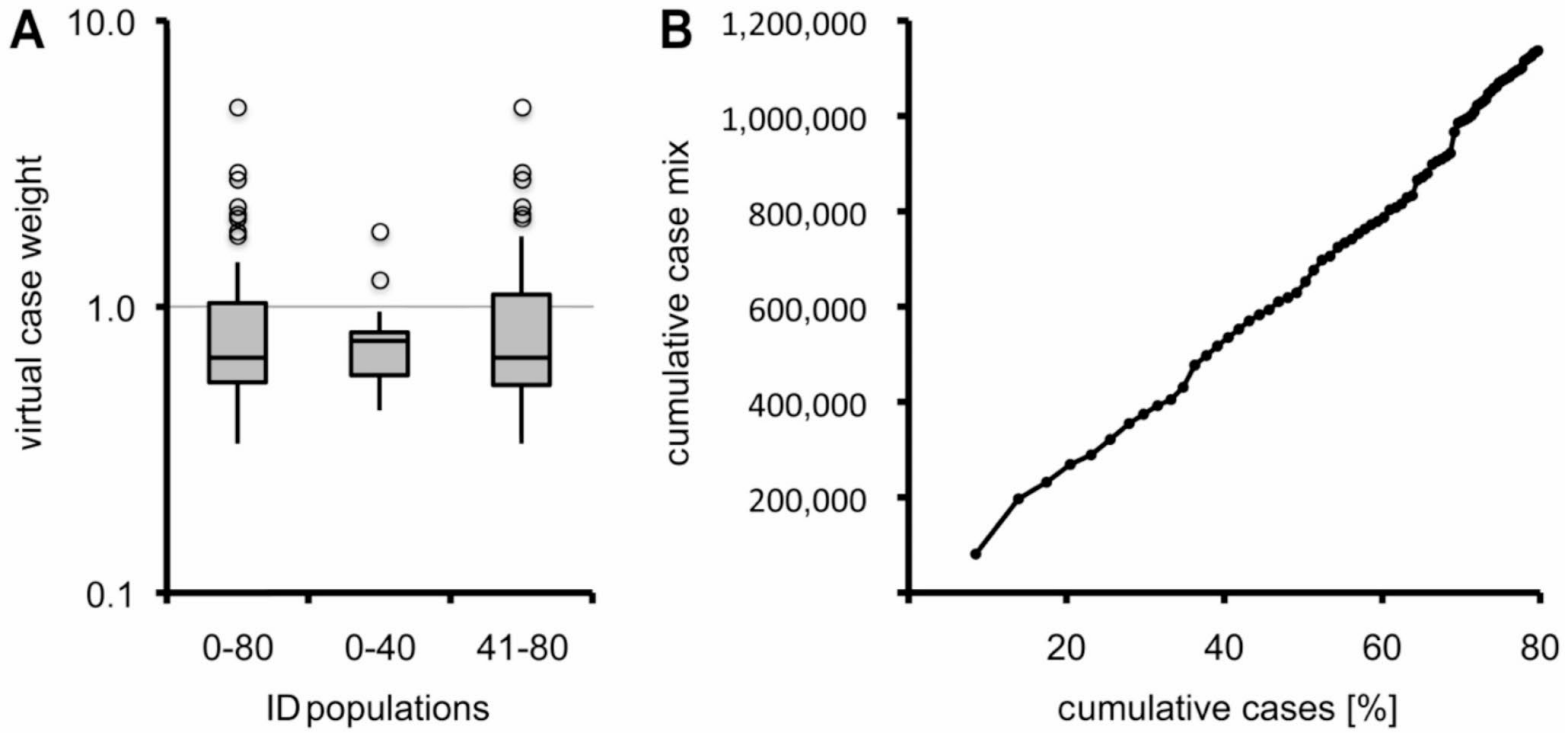
Virtual case weight distribution per ICD10 code and cumulative case mix per cumulative cases. Box plots of virtual case weights in the top 80% ID, the top 40% ID and the top 40–80% ID populations (panel A). Statistical high outliers were identified in each group. The high outlying ICD10 codes (description; cost weight) were the following: A41.0 (sepsis due to *Staphylococcus aureus*; 2.031), I33.0 (acute and subacute infective endocarditis; 4.967), J12.8 (other viral pneumonia; 1.237), J86.9 (pyothorax without fistula; 2.230), K57.22 (diverticulitis of large intestine with perforation and abscess; 1.827), M46.46 (discitis, unspecified, lumbar region; 2.775), T82.7 (infection and inflammatory reaction due to other cardiac and vascular devices, implants and grafts; 1.765), T84.5 (infection and inflammatory reaction due to internal joint prosthesis; 2.937) and T84.6 (infection and inflammatory reaction due to internal fixation device; 2.096). Except for A41.0 and J12.8, all other high outlying ICD10 codes can be allocated to the disciplines of surgery, orthopedics and cardiac surgery. Cumulative case mix per cumulative cases of the top 80% ID population (panel B)

## Electronic supplementary material

Below is the link to the electronic supplementary material.

Supplementary Material 1

## Data Availability

No datasets were generated or analysed during the current study.

## References

[1] Armstrong GL, Conn LA, Pinner RW. Trends in infectious disease mortality in the united States during the 20th century. JAMA. 1999;281(1):61–6. 9892452 10.1001/jama.281.1.61

[2] InEK DatenBrowser. In., vol. https://datenbrowser.inek.org: Institut für das Entgeltsystem im Krankenhaus GmbH (InEK).

[3] Budge S, Ambelu A, Bartram J, Brown J, Hutchings P. Environmental sanitation and the evolution of water, sanitation and hygiene. Bull World Health Organ. 2022;100(4):286–8. 35386561 10.2471/BLT.21.287137 PMC8958826

[4] Katchanov J, Wostmann K, Tominski D, Jefferys L, Liedtke A, Schneider A, Slevogt H, Arasteh K, Stocker H. Burden and spectrum of infectious disease in Germany 2009–2014: a multicentre study from Berlin’s municipal hospitals. Infection. 2016;44(2):187–95. 26311655 10.1007/s15010-015-0834-2

[5] Christensen KL, Holman RC, Steiner CA, Sejvar JJ, Stoll BJ, Schonberger LB. Infectious disease hospitalizations in the united States. Clin Infect Diseases: Official Publication Infect Dis Soc Am. 2009;49(7):1025–35. 10.1086/605562 19708796

[6] Meyer-Schwickerath C, Weber C, Hornuss D, Rieg S, Hitzenbichler F, Hagel S, Ankert J, Hennigs A, Glossmann J, Jung N. Complexity of patients with or without infectious disease consultation in tertiary-care hospitals in Germany. Infection. 2024;52(2):577–82. 38277092 10.1007/s15010-023-02166-w PMC10955003

[7] Bai AD, Showler A, Burry L, Steinberg M, Ricciuto DR, Fernandes T, Chiu A, Raybardhan S, Science M, Fernando E, et al. Impact of infectious disease consultation on quality of care, mortality, and length of stay in Staphylococcus aureus bacteremia: results from a large multicenter cohort study. Clin Infect Diseases: Official Publication Infect Dis Soc Am. 2015;60(10):1451–61. 10.1093/cid/civ120 25701854

[8] Chiong F, Wasef MS, Liew KC, Cowan R, Tsai D, Lee YP, Croft L, Harris O, Gwini SM, Athan E. The impact of infectious diseases consultation on the management and outcomes of Pseudomonas aeruginosa bacteraemia in adults: a retrospective cohort study. BMC Infect Dis. 2021;21(1):671. 34243714 10.1186/s12879-021-06372-5 PMC8268285

