# Supplementary material for: Caseload, clinical spectrum and economic burden of infectious diseases in patients discharged from hospitals in Germany
Source: Infection. 2025 Apr 9;53(5):1863–73. doi: 10.1007/s15010-025-02507-x (PMC12460425; doi:10.1007/s15010-025-02507-x)
Supplement: Supplementary file 1 — Supplementary Material 1 [file 15010_2025_2507_MOESM1_ESM.docx]

**Caseload, Clinical Spectrum and Economic Burden of Infectious Diseases in Patients discharged from Hospitals in Germany**

Stocker H^1,2^, Kron F^3,4,5^, Hartmann P^6^ de With K^7^, Addo M^8^, Vehreschild M^9^, Fätkenheuer G^10^, Salzberger B^11^, Sander LE^2^, Rupp J^12,13^

^1^ Department of Infectious Diseases, St. Joseph Hospital, Berlin-Tempelhof, Germany

^2^ Department of Infectious Diseases, Respiratory and Critical Care Medicine, Charité - Universitätsmedizin Berlin, Berlin, Germany

^3^ Department I of Internal Medicine, University Hospital of Cologne, University of Cologne, Cologne, Germany

^4^ VITIS Healthcare Group, Cologne, Germany

^5^ FOM University of Applied Sciences, Essen, Germany

^6^ Department of Infectious Diseases, Hospital of the Cellites - St. Vinzenz, Cologne, Germany

^7^ Institute of Infectious Diseases, University Hospital Carl Gustav Carus at the Technical University, Dresden, Germany

^8^ German Center for Infection Research, University Medical Center Hamburg-Eppendorf, Hamburg, Germany

^9^ Goethe University Frankfurt, University Hospital, Department 2 of Internal Medicine, Infectious Diseases, Frankfurt am Main, Germany

^10^ Department I of Internal Medicine, Division of Infectious Diseases, Medical Faculty and University Hospital of Cologne, University of Cologne, Cologne, Germany

^11^ Department of Infection Prevention and Infectious Diseases, University Hospital Regensburg, Regensburg, Germany

^12^ Institute of Medical Microbiology, University Hospital Schleswig-Holstein/ Campus Lübeck and Kiel, Kiel, Germany

^13^ Infectious Disease Clinic, University of Lübeck and University Hospital Schleswig-Holstein/ Campus Lübeck, Lübeck, Germany

**Supplement:**

*Additional information on cost weights:*

The cost weights for each DRG are calculated by the InEK based on the information from representative hospitals where the expenses are recorded and analyzed at the individual case level. Each year, the value of a cost weight point is negotiated between the representatives of the payors (i.e. the health care insurance companies) and the providers of in-patient health care based on the available financial resources, the expected number of cases assigned to each DRG and external factors such as the inflation rate and energy prices. In 2022, a cost weight of 1.000 corresponded to 3,833.00 €.

*Additional information on virtual cost weights:*

To illustrate the mathematical operation used to derive the virtual cost weights, we provide an example for the fictional code X99.Z:

If this code i) is used for a total of 100 cases and ii) triggers three different fictional DRGs with case weights of DRG1: 0.500 (n=60), DRG2: 1.000 (n=22) and DRG3: 1.200 (n=18). The case mixes (case weight x n) for the code / DRG will then be 30,000 (0.500 x 60) for cases triggering DRG1, 22,000 (1.000 x 22) for cases triggering DRG2 and 21,600 (1.200 x 18) for cases triggering DRG3, adding up to 73,600. Dividing this case mix by the number of cases (n=100) we receive the virtual cost weight for our fictional code X99.Z of 0.736.

Due to truncations of the dataset, we could not perform this operation for all ICD10 codes classified as IDs. We therefore limited our analysis to the codes that contained in sum 80% of the most frequent cases with a principal ID diagnosis (top 80% ID population). Even this population contained four ICD10 codes for which this operation was not possible due to lack of data. These codes were N13.63; hydronephrosis with ureteral calculus obstruction with renal infection (n= 5,733), K65.09; other and unspecified acute peritonitis (n= 5,557), Z22.8; carrier of other infectious diseases (n= 5,481) and N13.65: Other unspecified hydronephrosis with renal infection (n= 4,607). For these cases we imputed the median virtual cost weight derived from the remaining dataset of the top 80% ID population.
